# Prognostic Impact of Sarcopenia and Radiotherapy in Patients With Advanced Gastric Cancer Treated With Anti-PD-1 Antibody

**DOI:** 10.3389/fimmu.2021.701668

**Published:** 2021-07-08

**Authors:** Nalee Kim, Jeong Il Yu, Do Hoon Lim, Jeeyun Lee, Seung Tae Kim, Jung Yong Hong, Won Ki Kang, Woo Kyoung Jeong, Kyoung-Mee Kim

**Affiliations:** ^1^ Department of Radiation Oncology, Samsung Medical Center, Sungkyunkwan University School of Medicine, Seoul, South Korea; ^2^ Department of Medicine, Samsung Medical Center, Sungkyunkwan University School of Medicine, Seoul, South Korea; ^3^ Department of Radiology, Samsung Medical Center, Sungkyunkwan University School of Medicine, Seoul, South Korea; ^4^ Department of Pathology, Samsung Medical Center, Sungkyunkwan University School of Medicine, Seoul, South Korea

**Keywords:** gastric cancer, immunotherapy, radiation therapy, sarcopenia, inflammation, prognosis

## Abstract

**Background:**

We explored the combined effects of sarcopenia (SAR) and radiotherapy (RT) on outcomes in patients with advanced gastric cancer (AGC) treated with immune-checkpoint blockade (ICB).

**Methods:**

Among 185 patients with AGC treated with ICB, we defined SAR as skeletal muscle index <49 cm2/m2 for men and <31 cm2/m2 for women; 93 patients met criteria. We defined high neutrophil-to-lymphocyte ratio (hNLR) as NLR≥3. Palliative RT was performed in 37 patients (20%) before ICB.

**Results:**

We frequently observed hNLR in patients with SAR (53% *vs.* 35%, p = 0.02). The median overall survival (OS) for the entire cohort was 5 months. Stratification by risk factors of SAR or hNLR revealed a significant difference in median OS (0 [N = 60] *vs.* 1 [N = 76] *vs.* 2 [N = 49]: 7.6 *vs.* 6.4 *vs.* 2.2 months, p < 0.001). Patients with microsatellite instability-high (MSI-H, N = 19) or Epstein-Barr virus (EBV)-positive tumors (N = 13) showed favorable outcomes compared to those with microsatellite stable (MSS, N = 142) tumors (median OS, not reached *vs.* 16.8 *vs.* 3.8 months, respectively). The benefit of RT was evident in patients with both SAR and hNLR (median OS, 3.1 *vs.* 1.3 months, p = 0.02) and MSS/EBV-negative tumor (median OS, 6.5 *vs.* 3.5 months, p = 0.03), but outcomes after RT in MSI-H tumor were not significantly different. In multivariable analysis, SAR/hNLR, molecular subtypes, and a history of RT were associated with OS (all p < 0.05).

**Conclusions:**

We demonstrated the negative predictive value of SAR/hNLR on outcomes after ICB for AGC, and the history of RT could overcome the negative impact of SAR/hNLR and the MSS/EBV-negative subtype.

## Introduction

With the emergence of immune checkpoint blockade (ICB) therapy being used in several malignancies, several prospective trials have demonstrated the efficacy and safety of ICB in patients with advanced gastric cancer (AGC) after initial treatment. Anti-programmed death-1 (PD-1) monoclonal antibodies, nivolumab and pembrolizumab, demonstrated promising results in phase II/III trials (1–4). The ATTRACTION-2 phase III trial provided improved survival outcomes with nivolumab (median survival 5.3 and 4.1 months for nivolumab and placebo groups, respectively) (1). The KEYNOTE-061 phase III trial demonstrated improved outcomes with pembrolizumab as second-line therapy for AGC but failed to meet the significance threshold; the median survival was 9.1 and 8.3 months for the pembrolizumab and paclitaxel groups, respectively (2).

Despite favorable outcomes following ICB, the response rate is often limited (11–25%) in the salvage setting, which prompted physicians to identify predictive ICB biomarkers (1–3). PD-L1 (programmed death-ligand 1) expression is a well-known potential ICB biomarker in other solid tumors (5). A subsequent analysis of the ATTRACTION-2 trial revealed that the survival benefit of nivolumab remains significant regardless of PD-L1 status (1). However, the KEYNOTE-059 phase II trial showed higher response rate and durable response of pembrolizumab in patients with PD-L1-positive tumors: overall response rates (ORR) of 22.7% and 8.6% for patients with PD-L1-positive and -negative tumors, respectively (3). In addition to PD-L1 status, mutational burden, including microsatellite instability, is suggested as a predictive response factor for anti-PD-L1 ICB treatment (6, 7). Furthermore, an Epstein-Barr virus (EBV)-positive tumor subtype exhibits prominent immune cell infiltration in the tumor microenvironment and genomic features encoding PD-L1, which could make it potentially sensitive to ICB (8).

Apart from PD-L1 status and molecular features of AGC, systemic inflammation status reflected by sarcopenia (SAR) or serum inflammatory markers is also regarded as a potential biomarker for patients treated with ICB (9–12). SAR, characterized by the depletion of skeletal muscle mass, is well recognized as a negative factor for immunity and is often observed in chronic diseases (13). As a local treatment, radiation therapy (RT) has an immune-stimulating effect by the induction and enhancement of tumoricidal innate and adaptive immune responses (14). Herein, we performed a retrospective analysis to assess the association between SAR/inflammation and treatment response and identify the impact of RT on for patients with AGC treated with ICB.

## Materials and Methods

### Patient Population

Upon receiving Institutional Review Board approval (IRB number 2020-12-135), we retrospectively reviewed the data of patients with AGC treated with ICB at Samsung Medical Center from March 2016 to June 2019. Patients were excluded if they were treated with adjuvant RT following curative surgery and if the follow-up period was less than 1 month. We conducted the study in accordance with the provisions of the Declaration of Helsinki and Good Clinical Practice guidelines. The requirement for informed consent was waived owing to the retrospective nature of the study. All authors had access to the study data and reviewed and approved the final manuscript.

### Treatment

#### Immunotherapy

All patients received ICB after first- (80 patients, 43.2%) or second-line or more (105 patients, 56.8%) systemic chemotherapy. Pembrolizumab (200 mg) was administered intravenously every 3 weeks for 24 months or until disease progression, unacceptable toxicity, or patient’s decision to withdraw (2, 6). Nivolumab was administered intravenously at a dose of 3 mg/kg once every 2 weeks until the patients experienced unacceptable toxicity and disease progression or refused treatment (8).

#### Radiation Therapy

Before ICB, 37 (20.0%) patients underwent RT for palliative purposes. As summarized in **Supplementary Table S1**, the most common sites for RT were the stomach (12 patients, 32.4%), followed by the para-aortic lymph node region (7 patients, 18.9%), and bone (7 patients, 18.9%). All RT planning was performed using 6–9 MV photon under planning computed tomography (CT). The median interval between ICB and RT was 7.3 (interquartile range [IQR] 0.4–19.4) months.

### Data Collection

#### Sarcopenia

Instead of dual-energy X-ray absorptiometry, abdominal CT before the first cycle of ICB administration was used to evaluate body composition. Using the in-house semi-automated software (https://sourceforge.net/projects/muscle-fat-area-measurement), the cross-sectional area (cm^2^) of the skeletal muscle at the L3 level was assessed (11, 12). The skeletal muscle index (SMI) was calculated as follows: SMI (cm^2^/m^2^) = cross-sectional area (cm^2^)/height^2^ (m^2^). SAR was defined as SMI <49 cm^2^/m^2^ for men and <31 cm^2^/m^2^ for women, according to the Korean-specific cut-off values for SAR (15).

#### Neutrophil-to-Lymphocyte Ratio

The neutrophil-to-lymphocyte ratio (NLR) at the first administration of ICB was calculated as follows: absolute neutrophil count/absolute lymphocyte count. High NLR (hNLR) was defined as NLR ≥3, which is a widely accepted criterion (16).

#### Molecular Category

Immunohistochemistry staining and assessment for MLH1 (antibody: ES05 clone; 1:100 dilution; Novocastra) and MSH2 (clone G219-1129; 1:500 dilution; Cell Marque) in formalin-fixed paraffin-embedded tissue sections were used for determining the MSI status, as previously described (6). The loss of MLH1 and/or MSH2 expression defines the MSI-H status. EBV was evaluated using in-situ hybridization for EBV-encoded small RNA (17). Based on these results, we categorized patients into 4 groups: MSI-H, EBV positive, MSS/EBV negative, and unknown.

#### PD-L1 Status

Immunohistochemistry staining for PD-L1 was performed using a Dako 22C3 pharmDx kit (Agilent Technologies, Santa Clara, CA, USA). PD-L1 expression was determined based on the combined positivity score and calculated by dividing the total number of PD-L1 stained cells by the total number of viable tumor cells, multiplied by 100 (6).

### Statistical Analysis

Treatment responses following ICB were assessed using the Response Evaluation Criteria in Solid Tumors version 1.1. The ORR was defined as complete and partial responses; disease control rate (DCR) was defined as complete response, partial response, and stable disease lasting for ≥6 months (4). Overall survival (OS) was calculated from the first day of ICB administration to the date of death or last follow-up. Pearson chi-squared or Mann-Whitney U tests were employed to compare categorical or continuous variables between patients with or without SAR. OS was estimated with the Kaplan-Meier method, and comparisons were performed with the log-rank test. We performed multivariable analyses using the Cox proportional hazard model to test the independent significance of prognostic factors statistically significant in univariable analyses. In all analyses, a two-sided p-value of <0.05, was considered statistically significant. For testing multicollinearity among statistically significant factors, we checked the variance inflation factors less than 5. All statistical analyses were performed using R (version 4.0.2; R Foundation for Statistical Computing, Vienna, Austria; http://www.R-project.org).

## Results

### Patient Characteristics

The baseline characteristics of the entire cohort are summarized in **Table 1**. There were 19, 13, and 142 patients with MSI-H tumors, EBV-positive tumors, and MSS/EBV-negative tumors, respectively. In addition, there were 49 patients (26.5%) with PD-L1 ≥ 1% in their tumors. More than half of the patients had peritoneal carcinomatosis and distant-organ (i.e., lung, liver, bone) metastasis at the time of ICB treatment. Nivolumab and pembrolizumab were administered to 81 and 104 patients, respectively.

**Table 1:** Patient characteristics stratified by sarcopenia status.

		Total (N=185)	Sarcopenia (+) (N=93)	Sarcopenia (-) (N=92)	P-value
Age, year		59 [51-69]	62 [55-70]	57 [47-67]	0.027
Sex	Male	120 (64.9)	85 (91.4)	35 (38.0)	<0.001
BMI, kg/m^2^		21.2 [18.9-23.2]	20.6 [18.2-22.0]	22.0 [19.8-24.8]	<0.001
Underweight (<18.5)		40 (21.6)	25 (26.9)	15 (16.3)	0.117
SMI, cm^2^/m^2^		41.8 [36.0-47.4]	40.7 [34.6-44.3]	44.8 [37.7-53.3]	<0.001
Pathology	Adenoca, MD	57 (30.8)	33 (35.5)	25 (27.2)	0.591
	Adenoca, PD	103 (55.7)	50 (53.8)	53 (57.6)	
	Signet ring cell	22 (11.9)	9 (9.7)	13 (14.1)	
	Neuroendocrine	3 (1.6)	1 (1.1)	2 (2.2)	
Molecular category	MSI-H	19 (10.3)	6 (6.5)	13 (14.1)	0.209
	EBV (+)	13 (7.0)	8 (8.6)	5 (5.4)	
	MSS/EBV (-)	142 (76.8)	75 (80.6)	67 (72.8)	
	Unknown	11 (5.9)	4 (4.3)	7 (7.6)	
PD-L1 status (22C3 CPS)	≥1%	49 (26.5)	24 (25.8)	25 (27.2)	0.756
	<1%	46 (24.9)	24 (25.8)	22 (23.9)	
	Unknown	90 (48.6)	45 (48.4)	45 (48.9)	
Number of metastatic sites		2 [1-3]	2 [1-3]	2 [1-3]	0.467
Peritoneal carcinomatosis		127 (68.6)	70 (75.3)	57 (62.0)	0.073
Distant organ metastasis		115 (62.2)	52 (55.9)	63 (68.5)	0.107
Previous curative surgery		62 (33.5)	30 (32.3)	32 (34.8)	0.835
Previous radiation therapy		37 (20.0)	18 (19.4)	19 (20.7)	0.971
Total dose		35.0 [25.0-36.0]	36.0 [30.0-36.0]	30.0 [22.0-36.0]	0.330
Fractional dose		3.0 [3.0- 4.0]	3.0 [3.0- 3.0]	3.5 [3.0- 7.5]	0.061
Immune-checkpoint blockade	Nivolumab	81 (43.8)	48 (51.6)	33 (35.9)	0.044
	Pembrolizumab	104 (56.2)	45 (48.4)	59 (64.1)	
White blood cell count (x 10^3^/μL)		6.26 [4.86-8.80]	7.40 [5.32-9.57]	5.54 [4.46-7.50]	0.001
ANC (x 10^3^/μL)		3.92 [2.72-5.92]	4.68 [3.45-6.58]	3.12 [2.46-5.12]	0.001
ALC (x 10^3^/μL)		1.48 [1.09-1.91]	1.48 [1.09-1.91]	1.52 [1.08-1.93]	0.925
NLR		2.6 [1.7- 4.5]	3.3 [2.0- 4.9]	2.3 [1.4- 3.8]	0.003
NLR≥3		81 (43.8)	49 (52.7)	32 (34.8)	0.021
Albumin (g/dL)		3.8 [3.3- 4.2]	3.8 [3.3- 4.1]	3.8 [3.4- 4.2]	0.329
Albumin<3.5 g/dL		51 (27.6)	26 (28.0)	25 (27.2)	1.000

Values are presented as the number of patients (%) or medians [interquartile range] BMI, body mass index; SMI, skeletal muscle index; Adenoca, adenocarcinoma; MD, moderately differentiated; PD, poorly differentiated; MSS, microsatellite stable; EBV, Epstein-Barr virus; MSI-H, microsatellite instability-high; PD-L1, programmed death-ligand 1; CPS, combined positive score; ANC, absolute neutrophil count; ALC, absolute lymphocyte count; NLR, neutrophil-lymphocyte ratio.

Among 185 patients, there were 93 and 23 patients with and without SAR, respectively (**Table 1**). Patients with SAR were older (median 62 years); had lower body mass index (median 20.6 kg/m2); and had higher white blood cell count, absolute neutrophil count, and NLR (median 3.3), compared to those without SAR (p < 0.05). Specifically, hNLR was more frequently observed in patients with SAR than in those without SAR (52.7% *vs*. 34.8%, p = 0.021). There was no difference in the molecular category of tumors, PD-L1 positivity, and disease extent between the two groups.

Additionally, patients treated with pembrolizumab showed less frequent SAR and had more MSI-H and PD-L1 positive tumor compared to those treated with nivolumab (**Supplementary Table S2**).

### Response Rate

Overall, the ORR and DCR were 16.8% and 22.7%, respectively (**Table 2**). Patients with MSI-H tumors showed higher ORR (63.2%) and DCR (73.7%), followed by those with EBV-positive tumors (ORR, 53.8%; DCR, 38.5%). In addition, ORR and DCR were higher in patients with PD-L1≥1% in tumors than in those with PD-L1<1% in tumors. Importantly, DCR was lower in patients with SAR than in those without SAR (15.1% *vs*. 30.4%, p = 0.020), and both ORR and DCR were significantly different according to the baseline hNLR (ORR, 19.6% *vs*. 3.1%; DCR, 26.1% **vs*.* 6.2%). Patients with a history of RT showed higher but not statistically significant ORR and DCR than those with no history of RT (ORR, 29.7% *vs*. 13.5%, p = 0.058; DCR, 29.7% *vs*. 20.9%, p = 0.357).

**Table 2:** Response rate of immune checkpoint blockade.

Variables		ORR^a^ (%)	*p*-value	DCR^b^ (%)	*p*-value
Entire		16.8		22.7	
Pathology	SRC	19.0	0.080	23.9	0.418
	non-SRC	0.0		13.6	
Molecular category	MSI-H	63.2	<0.001	73.7	<0.001
	EBV (+)	53.8		38.5	
	MSS/EBV (-)	8.5		14.1	
	Unknown	0.0		27.3	
PD-L1 status	≥1%	36.7	<0.001	40.8	0.002
(22C3 CPS)	<1%	6.5		15.2	
	Unknown	11.1		16.7	
RT history	No	13.5	0.058	20.9	0.357
	Yes	29.7		29.7	
ICB sequence	After 1^st^ line chemotherapy	16.2	0.531	22.5	1.000
	After 2^nd^ line or more chemotherapy	17.1		22.9	
ICB	Nivolumab	7.4	0.008	11.1	0.002
	Pembrolizumab	24.0		31.7	
Sarcopenia	No	19.6	0.494	30.4	0.020
	Yes	14.0		15.1	
NLR≥3	No	19.6	0.005	26.1	0.027
	Yes	3.1		6.2	

a Complete response + partial response

b Complete response + partial response + stable disease maintained for ≥6 months.

ORR, objective response rate; DCR, disease control rate; SRC, signet ring cell carcinoma; MSS, microsatellite stable; EBV, Epstein-Barr virus; MSI-H, microsatellite instability-high; PD-L1, programmed death-ligand 1; CPS, combined positive score; RT, radiation therapy; ICB, immune-checkpoint blockade; NLR, neutrophil-lymphocyte ratio.

### Survival Outcomes

**Figure 1:**
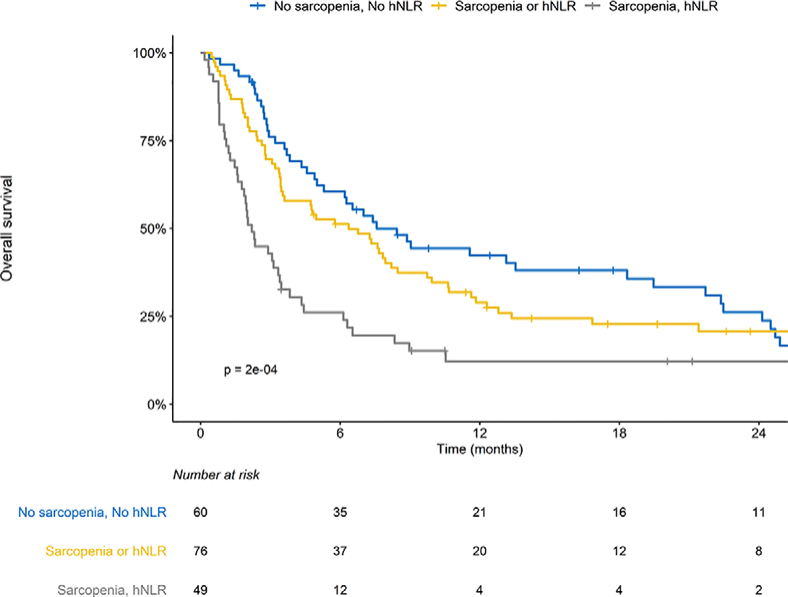
Overall survival (OS) stratified by sarcopenia (SAR) and high neutrophil-to-lymphocyte ratio (hNLR).

The median follow-up periods for all patients and the surviving patients were 4.8 (IQR, 2.2–11.6) months and 18.7 (IQR, 9.8–30.1) months, respectively. The median OS and 1-year OS rates were 4.9 months and 28.8%, respectively. Patients with SAR or hNLR exhibited inferior OS outcomes than those without SAR or hNLR (**Supplementary Figure S1**). Risk groups stratified by the presence of SAR and hNLR significantly differed in their OS outcomes; patients with both SAR and hNLR showed the worst OS outcomes (median OS: 0 **vs*.* 1 *vs*. 2 risk factors, 7.6 *vs*. 6.4 v. 2.2 months, p < 0.001, **Figure 1**). There was a significant difference in OS among molecular categories. The median OS in MSI-H positive, EBV-positive, and MSS/EBV-negative tumors was not reached yet, 16.8 months, and 3.8 months, respectively (p < 0.001, **Supplementary Figure S2**). Also, median OS for patients with PD-L1-positive tumors doubled compared with those with PD-L1-negative tumors (9.7 **vs*.* 4.9 months, p = 0.004, **Supplementary Figure S3**). After multivariable analysis, a history of RT (hazard ratio [HR] 0.47, 95% confidence interval [CI] 0.24-0.92, p = 0.028) and risk group stratification incorporating SAR and hNLR were associated with OS outcomes (**Table 3**). Additionally, older age (HR 0.58, 95% CI 0.35-0.95, p = 0.030) and MSI-H tumors were associated with favorable OS outcomes.

**Table 3:** Prognostic factors for overall survival.

Variables	(ref. *vs*. test)	Univariable analysis			Multivariable analysis		
		HR	95% CI	P-value	HR	95% CI	P-value
Age	(<60 *vs*. ≥ 60 yrs)	0.65	0.47-0.90	0.010	0.58	0.35-0.95	0.030
Pathology	(Non-SRC *vs*. SRC)	1.39	0.86-2.26	0.179			
BMI (kg/m^2^)	(≥18.5 *vs*. <18.5)	1.99	1.35-2.92	<0.001	1.79	0.98-3.28	0.058
Molecular category	MSS/EBV negative	Ref.			Ref.		
	MSI-H	0.18	0.08-0.40	<0.001	0.08	0.03-0.28	<0.001
	EBV positive	0.45	0.22-0.92	0.029	0.76	0.29-1.99	0.577
PD-L1 (22C3 CPS)	(<1% *vs*. ≥ 1%)	0.51	0.35-0.76	0.001	1.16	0.68-1.99	0.584
ICB	(Nivolumab*vs*. Pembrolizumab)	0.55	0.40-0.78	0.001	0.98	0.56-1.71	0.942
ICB sequence	(After 1^st^ line *vs*. 2^nd^ or more line)	0.79	0.57-1.09	0.153			
Metastatic sites	(<2 *vs*. ≥2)	1.49	1.03-2.17	0.035	1.53	0.87-2.66	0.138
Previous curative surgery	(No *vs*. yes)	0.78	0.55-1.11	0.164			
Radiation therapy	(No *vs*. yes)	0.62	0.40-0.96	0.033	0.47	0.24-0.92	0.028
Interval between radiation therapy and ICB	(<6 *vs*. ≥6 months)	0.81	0.63-1.04	0.092			
Baseline albumin	(≥3.5 *vs*. <3.5 g/dL)	2.16	1.50-3.11	<0.001	1.34	0.95-1.88	0.094
Number of risk factors(sarcopenia & NLR≥3)	0	Ref.			Ref.		
	1	1.23	0.83-1.82	0.300	2.02	1.09-3.74	0.026
	2	2.39	1.56-3.66	<0.001	6.06	3.04-12.08	<0.001

*The foreparts of parentheses are set as the reference group.

HR, hazard ratio; CI, confidence interval; SRC, signet ring cell carcinoma; BMI, body mass index; MSS, microsatellite stable; EBV, Epstein-Barr virus; MSI-H, microsatellite instability-high; PD-L1, programmed death-ligand 1; CPS, combined positive score; ICB, immune-checkpoint blockade; NLR, neutrophil-lymphocyte ratio.

### Impact of RT on Subgroup Analysis

**Figure 2:**
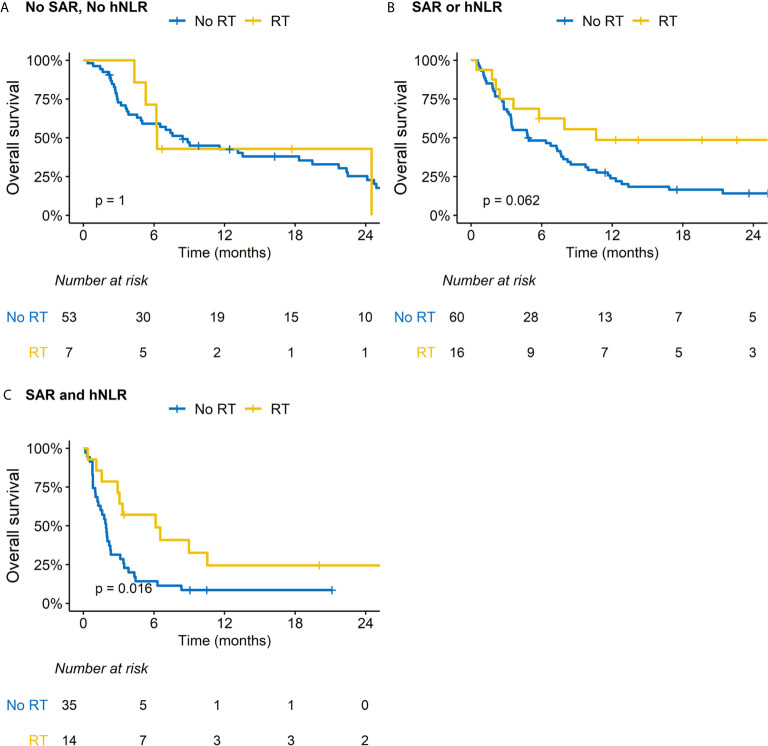
Impact of radiation therapy (RT) on overall survival according to subgroups based on sarcopenia (SAR) and high neutrophil-to-lymphocyte ratio (hNLR). **(A)** Patients with no SAR and hNLR; **(B)** Patients with SAR or hNLR; **(C)** Patients with SAR and hNLR.

**Figure 3:**
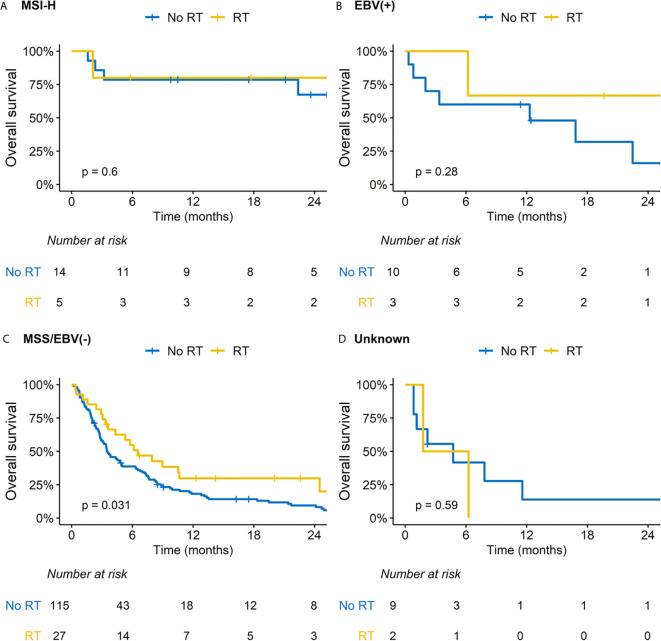
Impact of radiation therapy (RT) on overall survival according to subgroups based on molecular category. **(A)** Microsatellite instability-high (MSI-H) tumor; **(B)** Ebstein-Barr virus (EBV) positive tumor; **(C)** Microsatellite stable (MSS)/EBV negative tumor; **(D)** Unknown.

We performed subsequent subgroup analysis according to RT because this was related with improved OS outcomes in the multivariable analysis. Patients treated with RT had less frequent peritoneal seeding but more frequent distant visceral organ metastasis; other baseline characteristics were comparable between two groups (**Supplementary Table S3**). Regarding ORR and DCR, RT significantly benefitted patients with MSS/EBV-negative tumors and those with both SAR and hNLR (**Supplementary Table S4**). There was a difference in the impact of RT on OS outcomes for patients stratified by SAR and hNLR (**Figures 2A–C**). Specifically, although a borderline difference in OS according to RT was observed in patients with either SAR or hNLR (**Figure 2B**), RT significantly improved OS outcomes in patients with both SAR and hNLR (median OS: 3.1 *vs*. 1.3 months, p = 0.016, **Figure 2C**). Additionally, there was no significant difference in OS outcomes by RT in patients with favorable molecular categories (MSI-H or EBV-positive tumors, **Figures 3A, B**). In contrast, RT was associated with superior OS outcomes in patients with MSS/EBV-negative tumors (median OS: 6.5 *vs*. 3.5 months, p = 0.031, **Figure 3C**). However, RT had little impact on OS outcomes in the subgroup analysis based on PD-L1 status (**Supplementary Figures S4A–C**).

## Discussion

In the current study, we observed that CT-determined SAR at baseline was associated with frequent hNLR and that risk group stratification based on SAR and hNLR could be a potential surrogate for predicting outcomes in AGC patients treated with ICB, independent of previously identified molecular biomarkers, including MSI-H and/or EBV positivity. Additionally, RT improved outcomes in patients with unfavorable features, such as SAR/hNLR or MSS/EBV-negative tumors.

SAR in gastric cancer negatively affects postoperative morbidity and mortality for surgically resected patients; it also leads to poor OS outcomes in patients with advanced disease treated with chemotherapy (18–20). Recently, Kim et al. demonstrated that CT-determined SAR was associated with inferior results after ICB (12). They investigated 149 patients with MSS type gastric cancer and reported that SAR was related to shorter OS (median 3.6 *vs.* 4.9 months), but it was not statistically significant in multivariable analysis. Consistent with this report, we also observed a negative impact of SAR on survival outcomes for ICB-treated AGC patients. Additionally, we revealed that hNLR, frequently observed in patients with SAR, could be incorporated into risk group stratification for patients with ICB-treated AGC. Cytokines related to T-cell exhaustion, transforming growth factor-β, and interleukin-6 are known to be related to the development of SAR, resulting in reduced ICB efficacy in patients with SAR (9, 21–24). Furthermore, under the condition of SAR with skeletal muscle loss, impaired myokine (i.e., interleukin-15 and -16) signaling induces immune dysregulation and a proinflammatory environment (25, 26). This detrimental effect of SAR/hNLR has been widely investigated in patients with other solid tumors treated with ICB. Increased levels of interleukin-6 or transforming growth factor-β related to skeletal muscle atrophy contributed to poor treatment response in malignant melanoma, non-small cell lung cancer, and urothelial cancer treated with ICB (24, 27, 28).

In our analysis, RT was significantly associated with improved outcomes in patients with SAR/hNLR or MSS/EBV-negative tumors. Furthermore, after multivariable analysis, the impact of RT was statistically significant. A previous molecular study of ICB-treated AGC patients reported that MSI-H and EBV positivity could be reliable biomarkers for ICB in AGC patients (6, 7). Kim et al. reported remarkable responses to pembrolizumab: ORR of 85.7% in 7 patients with MSI-H tumors and 100% in 6 patients with EBV-positive tumors (6). Also, Mishima et al. also reported improved ORR of 75.0% in 8 patients with mismatch-repair-deficient tumors (7). We also observed higher OS rates for patients with MSI-H or EBV-positive tumors. However, we firstly observed a positive impact of RT in patients with MSS/EBV-negative tumors. Unlike immunogenic features of MSI-H tumor, immunogenic-cold features of MSS might lead to reduced efficacy of ICB (29). Similar to AGC, MSI-H is a well-established predictive biomarker for ICB in colorectal cancer (30). For overcoming resistance to ICB in MSS tumors, various strategies combining chemotherapy, a tyrosine kinase inhibitor, RT, and molecular target agents are now under clinical investigations (31, 32). Among these strategies, a growing body of evidence indicates that RT has a role in immunomodulation in the tumor microenvironment (14, 33, 34). Increased dendritic cell activation and T-cell priming through chemokines of CXC Chemokine Ligand 9-11 and -16, and macrophage differentiation could promote T-cells to invade into the tumor microenvironment (33). This immunologic dynamic after RT might transform immunologically cold-tumors into hot-tumors, potentially leading to improved outcomes for patients with SAR/hNLR or MSS/EBV-negative tumors (35). Here, we first demonstrated the preliminary clinical results of the positive impact of RT in those patients, supporting further clinical investigations. Additionally, the underlying mechanism of how RT improves outcomes in these patients (i.e., SAR/hNLR or MSS/EBV negative tumor) needs further preclinical investigations.

There are several limitations to be acknowledged. First, owing to the limited number of patients receiving RT before ICB, subgroup analyses based on RT had limited statistical power. Moreover, PD-L1 status was not available for 90 patients (48.6%). In this context, we did not observe a statistical significance of RT in patients with PD-L1-positive tumors (**Supplementary Figure S4B**). Given the high rates of early disease progression or death (in most patients) within 6 months, following ICB administration, SMI or lymphocyte/neutrophil counts at post-ICB administration could not be analyzed. In addition, the median interval between ICB and RT was relatively longer to identify the direct impact of RT in an immune modulation. However, Moravan et al. showed long-lasting immune-modulatory effect of RT in brain immune cells with elevated mature dendritic cells after 1 year of RT (36). In addition, RT induced immunogenic cell death could provide long-term immunological memory resulting in the priming of the immune system which could last long time (37, 38). This study is hypothesis-generating for the clinical significance of SAR/hNLR and the positive impact of RT in patients with unfavorable factors. A further preclinical study investigating concurrent or sequential RT with ICB administration could address the underlying pathway between RT and the tumor microenvironment in patients with SAR/hNLR or MSS/EBV-negative tumors. However, to the best of our knowledge, this is the first study to identify the prognostic factors for response after ICB, incorporating SAR/hNLR and the history of RT in patients with AGC who underwent ICB.

In summary, we suggest that RT might overcome the negative impact of risk factors including SAR/hNLR and MSS/EBV-negative tumors in ICB-treated patients with AGC. Considering the cost-effectiveness of ICB, baseline SAR, hNLR, and history of RT in addition to the molecular nature of the tumor (i.e., MSI-H, MSS, EBV, and PD-L1 status) could be considered for proper patient selection in clinical practice.

## Data Availability Statement

The raw data supporting the conclusions of this article will be made available by the authors, without undue reservation.

## Ethics Statement

The studies involving human participants were reviewed and approved by Samsung Medical Center. Written informed consent for participation was not required for this study in accordance with the national legislation and the institutional requirements.

## Author Contributions

Conception, design, data collection, interpretation, and drafting of the manuscript were performed by NK, JY, JL, and WJ. Data collection and interpretation were performed by NK, JY, DL, and WJ. Statistical analysis and editing of the manuscript were performed by NK, JY, JL, and WJ. All authors contributed to the article and approved the submitted version.

## Funding

This research was funded by a Basic Science Research Program through the National Research Foundation of Korea (NRF) funded, grant number NRF-2020R1D1A1B03031275.

## Conflict of Interest

The authors declare that the research was conducted in the absence of any commercial or financial relationships that could be construed as a potential conflict of interest.

## Abbreviations

ICB, immune-checkpoint blockade; AGC, advanced gastric cancer; PD-1, programmed death-1; PD-L1, programmed death-ligand 1; EBV, Epstein-Barr virus; SAR, sarcopenia; RT, radiation therapy; IQR, interquartile range; CT, computed tomography; SMI, skeletal muscle index; NLR, neutrophil-to-lymphocyte ratio; hNLR, high neutrophil-to-lymphocyte ratio; OS, overall survival; ORR, overall response rate; DCR, disease control rate; HR, hazards ratio; CI, confidence interval.
