# Supplementary material for: Prognostic Impact of Sarcopenia and Radiotherapy in Patients With Advanced Gastric Cancer Treated With Anti-PD-1 Antibody
Source: Front Immunol. 2021 Jul 8;12:701668. doi: 10.3389/fimmu.2021.701668 (PMC8298191; doi:10.3389/fimmu.2021.701668)
Supplement: Supplementary file 1 [file DataSheet_1.docx]

Supplementary Material

**
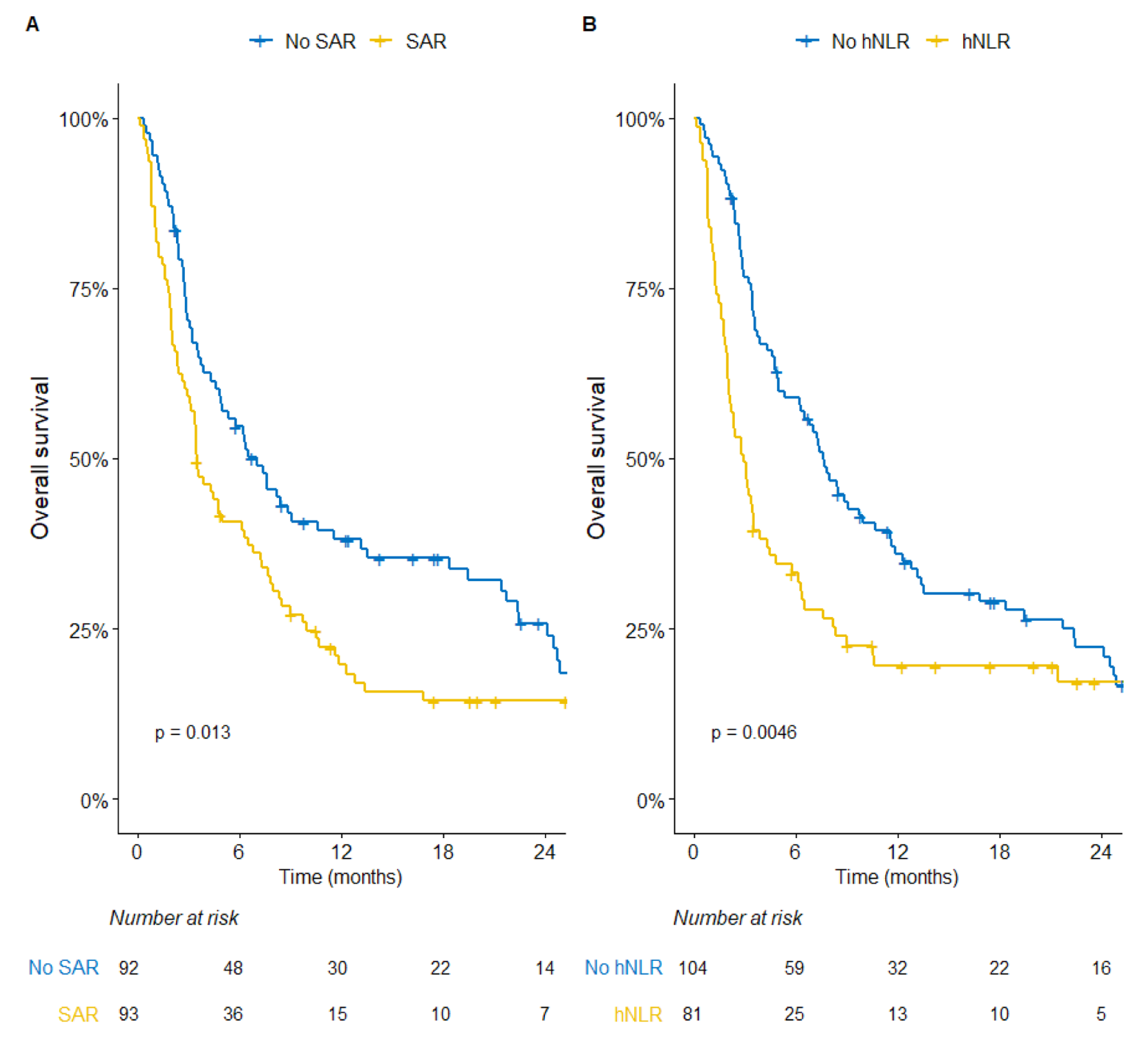
**

**Supplementary Figure 1.** Overall survival according to sarcopenia (A) and high neutrophil-to-lymphocyte ratio (B).

**
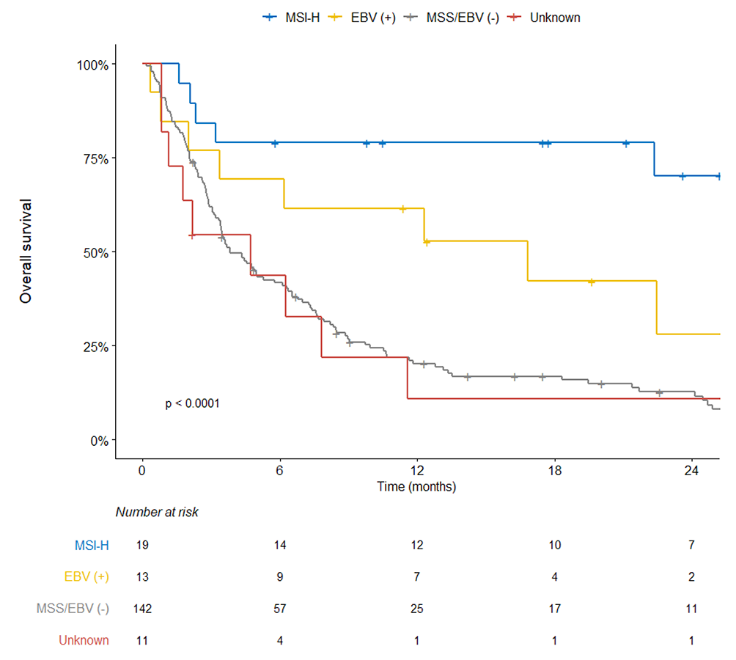
**

**Supplementary Figure 2.** Overall survival according to the molecular category

Footnotes: MSS, microsatellite stable; EBV, Epstein-Barr virus; MSI-H, microsatellite instability-high;

**
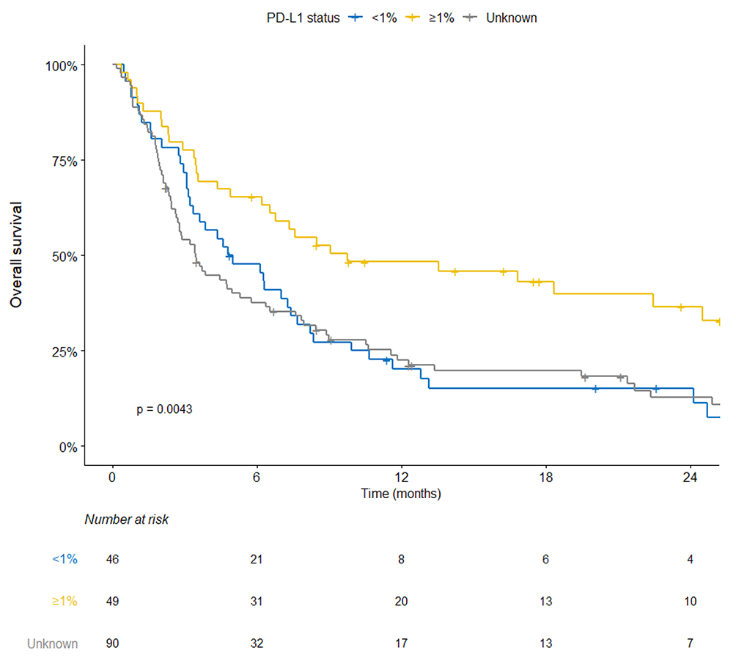
**

**Supplementary Figure 3.** Overall survival according to programmed death-ligand 1 (PD-L1) status

**
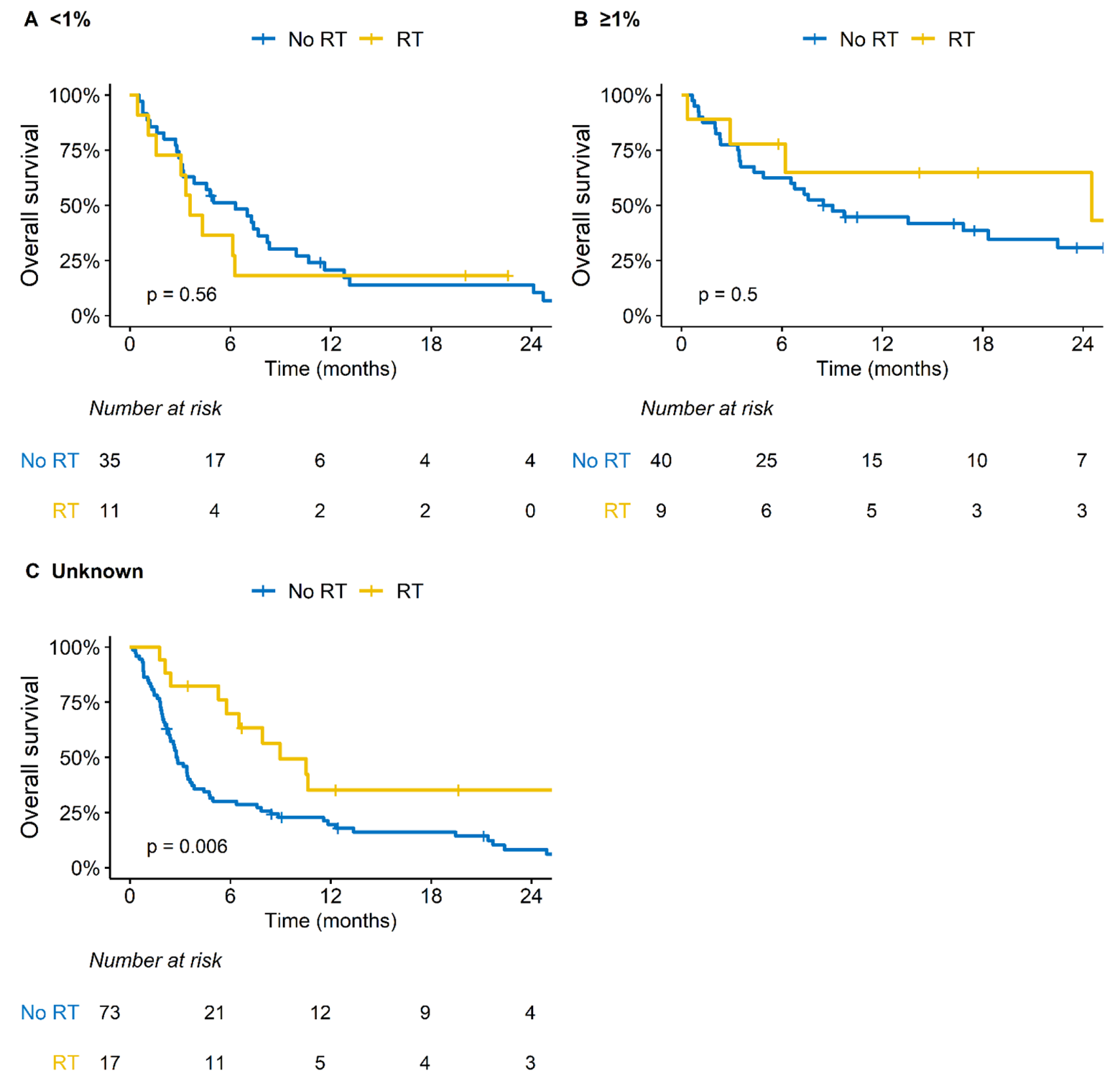
**

**Supplementary Figure 3.** Impact of radiation therapy (RT) on overall survival according to subgroups based on programmed death-ligand 1 (PD-L1) status

**Supplementary Table S1 |** Treatment details of radiation therapy.

| Interval between ICB and radiation therapy, months |  | Median [IQR]  7.3 [0.4-19.4]  N (%) |
| --- | --- | --- |
| Sequence of radiation therapy | Concurrent | 6 (16.2) |
|  | Before ICB | 31 (83.8) |
| Site | Stomach | 12 (32.4) |
|  | Paraaortic lymph node region | 7 (18.9) |
|  | Bone | 7 (18.9) |
|  | Pelvic seeding nodules | 5 (13.5) |
|  | Liver | 4 (10.8) |
|  | Pancreas | 1 (2.7) |
|  | Lung | 1 (2.7) |

*Abbreviations: IQR, interquartile range; ICB, immune-checkpoint blockade*

**Supplementary Table S2 |** Patient characteristics stratified by the immune-checkpoint blockade.

|  |  | **Nivolumab**  **(N=81)** | **Pembrolizumab**  **(N=104)** | **P-value** |
| --- | --- | --- | --- | --- |
| Age, year |  | 60 [53–69] | 69 [48–70] | 0.614 |
| Sex | Male | 56 (69.1) | 64 (61.5) | 0.358 |
| BMI, kg/m^2^ |  | 20.8 [18.3-23.5] | 21.3 [19.2-23.1] | 0.360 |
| Underweight (<18.5) |  | 23 (28.4) | 17 (16.3) | 0.073 |
| SMI, cm^2^/m^2^ |  | 40.9 [34.9-45.5] | 42.6 [37.9-48.9] | 0.178 |
| SAR |  | 48 (59.3) | 45 (43.3) | 0.044 |
| Molecular category | MSI-H | 0 (0.0) | 19 (18.3) | <.001 |
|  | EBV (+) | 5 (6.2) | 8 (7.7) |  |
|  | MSS/EBV (-) | 69 (85.2) | 73 (70.2) |  |
|  | Unknown | 7 (8.6) | 4 (3.8) |  |
| PD-L1 status (22C3 CPS) | ≥1% | 17 (21.0) | 32 (30.8) | 0.039 |
|  | <1% | 16 (19.8) | 30 (28.8) |  |
|  | Unknown | 48 (59.3) | 42 (40.4) |  |
| ANC (x 10^3^/μL) |  | 3.92 [2.74 -5.99] | 3.88 [2.72-5.85] | 0.957 |
| ALC (x 10^3^/μL) |  | 1.41 [1.04-1.86] | 1.60 [1.12-1.98] | 0.021 |
| NLR |  | 3.09 [1.95-4.65] | 2.50 [1.63-4.23] | 0.136 |
| NLR≥3 |  | 41 (50.6) | 40 (38.5) | 0.133 |

*Values are presented as the number of patients (%) or medians [interquartile range]
Abbreviations: BMI, body mass index; SMI, skeletal muscle index; SAR, sarcopenia; MSS, microsatellite stable; EBV, Epstein-Barr virus; MSI-H, microsatellite instability-high; PD-L1, programmed death-ligand 1; CPS, combined positive score;* *ANC, absolute neutrophil count; ALC, absolute lymphocyte count; NLR, neutrophil-lymphocyte ratio.*

**Supplementary Table S3 |** Baseline characteristics according to the history of radiation therapy (RT).

|  |  | **RT**  **N=37** | **No RT**  **N=148** | **P-value** |
| --- | --- | --- | --- | --- |
| Age, year |  | 59 [50-70] | 59 [52-69] | 0.478 |
| Sex | Male | 13 (35.1) | 52 (35.1) | 1.000 |
| BMI, kg/m^2^ |  | 21.4 [18.5-24.1] | 21.0 [18.9-23.2] | 0.879 |
| Underweight (<18.5) |  | 10 (27.0) | 30 (20.3) | 0.503 |
| SMI, cm^2^/m^2^ |  | 45.3 [36.7-47.1] | 41.4 [35.9-47.4] | 0.814 |
| SAR |  | 18 (48.7) | 75 (50.7) | 0.971 |
| Pathology | SRC | 3 (8.1) | 19 (12.8) | 0.609 |
|  | Non-SRC | 34 (91.9) | 129 (87.2) |  |
| Peritoneal seeding |  | 19 (51.4) | 108 (73.0) | 0.019 |
| Distant metastasis |  | 28 (75.7) | 87 (58.8) | 0.048 |
| Number of metastatic sites |  | 2 [2-3] | 2 [1-3] | 0.516 |
|  | ≥2 | 28 (75.7) | 87 (58.8) | 0.088 |
| Molecular category | MSI-H | 5 (13.5) | 14 (9.5) | 0.883 |
|  | EBV (+) | 3 (8.1) | 10 (6.8) |  |
|  | MSS/EBV (-) | 27 (73.0) | 115 (77.7) |  |
|  | Unknown | 2 (5.4) | 9 (6.1) |  |
| PD-L1 status (22C3 CPS) | ≥1% | 9 (24.3) | 40 (27.0) | 0.744 |
|  | <1% | 11 (29.7) | 35 (23.7) |  |
|  | Unknown | 17 (46.0) | 73 (49.3) |  |
| NLR≥3 & Sarcopenia | 0 | 7 (18.9) | 53 (35.8) | 0.086 |
|  | 1 | 16 (43.2) | 60 (40.5) |  |
|  | 2 | 14 (37.8) | 35 (23.7) |  |

*Values are presented as the number of patients (%) or medians [interquartile range]
Abbreviations: BMI, body mass index; SMI, skeletal muscle index; SAR, sarcopenia; MSS, microsatellite stable; EBV, Epstein-Barr virus; MSI-H, microsatellite instability-high; PD-L1, programmed death-ligand 1; CPS, combined positive score; NLR, neutrophil-lymphocyte ratio.*

**Supplementary Table S4 |** Response rate according to the history of radiation therapy (RT).

| **Overall response rate^a^ (%)** |  | **RT** | **no RT** | ***p*-value** |
| --- | --- | --- | --- | --- |
| Pathology | SRC | 0.0 | 0.0 | NA |
|  | non-SRC | 32.4 | 15.5 | 0.082 |
| Molecular category | MSI-H | 60.0 | 64.3 | 1.000 |
|  | EBV (+) | 66.7 | 50.0 | 0.800 |
|  | MSS/EBV (-) | 22.2 | 5.2 | 0.017 |
|  | Unknown | 0.0 | 0.0 | NA |
| PD-L1 status | ≥1% | 55.6 | 32.5 | 0.162 |
| (22C3 CPS) | <1% | 9.1 | 5.7 | 0.794 |
|  | Unknown | 29.4 | 6.8 | 0.019 |
| ICB | Nivolumab | 16.7 | 4.8 | 0.127 |
|  | Pembrolizumab | 42.1 | 20.0 | 0.084 |
| NLR≥3 & Sarcopenia | 0 | 28.6 | 17.0 | 0.682 |
|  | 1 | 37.5 | 16.7 | 0.177 |
|  | 2 | 21.4 | 2.9 | 0.038 |
| **Disease control rate^b^ (%)** |  | **RT** | **no RT** | ***p*-value** |
| Pathology | SRC | 0.0 | 15.8 | 1.000 |
|  | non-SRC | 32.4 | 21.7 | 0.285 |
| Molecular category | MSI-H | 60.0 | 78.6 | 0.827 |
|  | EBV (+) | 66.7 | 30.0 | 0.640 |
|  | MSS/EBV (-) | 22.2 | 10.2 | 0.028 |
|  | Unknown | 0.0 | 33.3 | 0.936 |
| PD-L1 status | ≥1% | 44.4 | 40.0 | 1.000 |
| (22C3 CPS) | <1% | 9.1 | 17.1 | 0.867 |
|  | Unknown | 35.3 | 12.3 | 0.054 |
| ICB | Nivolumab | 16.7 | 9.5 | 0.671 |
|  | Pembrolizumab | 42.1 | 29.4 | 0.422 |
| NLR≥3 & Sarcopenia | 0 | 28.6 | 30.2 | 1.000 |
|  | 1 | 43.8 | 21.7 | 0.143 |
|  | 2 | 14.3 | 5.7 | 0.045 |

*^a^Complete response + partial response*

*^b^Complete response + partial response + stable disease maintained for ≥6 months*

*Abbreviations: SRC, signet ring cell carcinoma; MSS, microsatellite stable; EBV, Epstein-Barr virus; MSI-H, microsatellite instability-high; PD-L1, programmed death-ligand 1; CPS, combined positive score; RT, radiation therapy; ICB, immune-checkpoint blockade; NLR, neutrophil-lymphocyte ratio.*
